# A modern circadian clock in the common angiosperm ancestor of monocots and eudicots

**DOI:** 10.1186/1741-7007-8-55

**Published:** 2010-05-07

**Authors:** C Robertson McClung

**Affiliations:** 1 Department of Biological Sciences, 6044 Gilman Laboratories, Dartmouth College, Hanover, New Hampshire 03755, USA

## Abstract

The circadian clock enhances fitness through temporal organization of plant gene expression, metabolism and physiology. Two recent studies, one in *BMC Evolutionary Biology*, demonstrate through phylogenetic analysis of the *CCA1*/*LHY *and *TOC1*/*PRR *gene families that the common ancestor of monocots and eudicots had components sufficient to construct a circadian clock consisting of multiple interlocked feedback loops.

See research article http://www.biomedcentral.com/1471-2148/10/126

## 

The recent availability of several plant genome sequences has made it clear that whole genome duplication (polyploidization) has occurred frequently during angiosperm evolution. It is thought that the provision of duplicated genes permits evolution through functional specialization as well as the acquisition of innovative functions. There are several examples in which multiple members of gene families contribute to the circadian clock mechanism, raising a number of questions. Practically, functional redundancy among family members limits the identification of clock components through forward genetics [1]. Of more general interest is the question of how these gene families have evolved among plants. In addition, there is considerable interest in determining the extent to which the clock model that has been developed for *Arabidopsis *will serve as a model for clock function among plants in general. A recent paper in *BMC Evolutionary Biology *describing the angiosperm *PSEUDO-RESPONSE REGULATOR *(*PRR*) gene family addresses each of these questions [2].

## Circadian clocks: complex and highly conserved mechanisms for coordinating metabolism and physiology with the environment

A circadian rhythm is an endogenously generated rhythm with a period of about 24 h, approximating the period of the rotation of the earth on its axis. These rhythms provide temporal organization of biological processes from cyanobacteria to mammals [3]. In plants, circadian rhythmicity is widespread and pervasive [4,5]. Approximately one-third of the *Arabidopsis *transcriptome shows circadian oscillations in abundance in continuous conditions [6], but if one looks under a variety of light and temperature cycles that proportion grows to an astonishing ~90% [7], underlining the probable importance of circadian rhythm to overall fitness [4,5].

**Figure 1:**
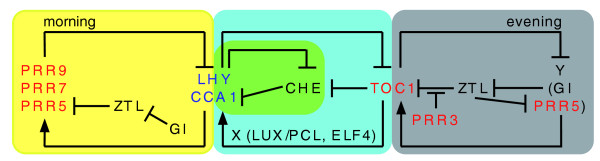
**Model of the *Arabidopsis *clock, emphasizing the roles of the *TOC1*/*PRR *(red) and *CCA1*/*LHY *(blue) genes**. The model is oversimplified to illustrate the interlocked feedback loops. Not all known clock components are included and undoubtedly more components remain unidentified. The central loop of *CCA1*, *LHY*, *TOC1*, and 'X' is shaded blue. The second loop of *CCA1 *and *CHE *is shaded green. The third ('morning') loop of *CCA1*, *LHY*, *PRR9*, *PRR7*, and *PRR5 *is shaded yellow, and the fourth ('evening') loop of *TOC1 *and 'Y' is shaded gray.

Circadian clocks of taxonomic groups as diverse as plants, fungi and animals are composed of multiple interlocked feedback loops with positive and negative components [3] and many of the components of these clocks are encoded by members of gene families. The *Arabidopsis *circadian clock, an example of this common design principle, is composed of at least four interlocked feedback loops (Figure 1). In the central loop (blue in Figure 1), *TIMING OF CAB EXPRESSION 1 *(*TOC1*), the founding member of a family of five *PSEUDO-RESPONSE REGULATOR *(*PRR*) genes, is a positive regulator of *CIRCADIAN AND CLOCK ASSOCIATED 1 *(*CCA1*) and *LATE ELONGATED HYPOCOTYL *(*LHY*). *CCA1 *and *LHY *are members of a small family of *REVEILLE *genes that encode single Myb domain transcription factors. Others members of this family have been shown to play roles in clock function as well as in regulation of clock output pathways [4,5]. To complete the first loop, CCA1 and LHY bind to the *TOC1 *promoter to inhibit its expression. In a second loop (green in Figure 1) within the central loop, CCA1 and LHY also repress expression of *CCA1 HIKING EXPEDITION *(*CHE*), which encodes a TCP transcription factor that binds to and represses expression from the *CCA1 *promoter [1]. In the third loop (yellow in Figure 1), termed the 'morning' loop based on the time of peak mRNA accumulation of its constituents, CCA1 and LHY are positive regulators of two *TOC1 *relatives, *PRR7 *and *PRR9*, that are negative regulators of *CCA1 *and *LHY *[4,5]. In a fourth loop (gray in Figure 1), termed the 'evening' loop based again on the time of peak mRNA accumulation, TOC1 represses a component, 'Y', that includes GIGANTEA (GI) and possibly PRR5. This component in turn positively regulates *TOC1 *expression at least in part through modulation by GI of proteasomal degradation of TOC1 mediated by the F-box protein ZEITLUPE (ZTL) [4,5]. In addition, proper regulation of *CCA1 *and *LHY *requires other clock genes, including *EARLY FLOWERING 4 *(*ELF4*), which encodes a protein of unknown function, and *LUX ARRHYTHMO*/*PHYTOCLOCK1 *(*LUX*/*PCL*), which encodes a Myb domain transcription factor; these and other clock components have yet to be fully incorporated into current clock models [4,5]. The number of interlocked feedback loops will undoubtedly increase as the regulatory relationships among clock components are more fully described.

The value of model organisms such as *Arabidopsis *stems from the generalization of knowledge acquired in the model to all flowering plants, and especially to those of agricultural significance. The increasing availability of genomic sequences from multiple plants is now permitting our first insights into this issue.

## Phylogenetic analysis of the PRR and *CCA1*/*LHY *gene families shows that circadian clocks composed of multiple interlocked feedback loops evolved prior to the divergence of monocots and eudicots

Molecular phylogenetic analysis of the *PRR *genes indicates that the common ancestor of the monocots and eudicots had three *PRR *gene clades [2]. Since the divergence of the monocots and eudicots, the clades corresponding to *PRR3/PRR7 *and *PRR5/PRR9 *have expanded independently in both lineages as a result of genome duplications [2]. Within the eudicots, or 'true dicots', a subset of the former broad classification of dicots that includes more than half of extant plant species, two further genome duplications occurred in *Arabidopsis *following its divergence from papaya (*Carica papaya*) but, after each duplication, one of the paralogs was lost. In contrast, poplar has retained the duplicate copies of *PRR5*, *PRR7 *and *PRR9*, which originated in a genome duplication, termed the Salicoid duplication, that occurred in the poplar lineage after its separation from the papaya-*Arabidopsis *lineage. *PRR3 *has been completely lost from the poplar genome, although it is unclear whether this loss predated or followed the Salicoid duplication. The *Brassica rap*a genome has triploidized since its divergence form *Arabidopsis *approximately 14.5 million years ago, yet for no members of the *B. rapa TOC1*/*PRR *gene family have all three copies persisted, making it clear that differential *PRR *gene loss has occurred [8].

Takata *et al. *[9] have conducted a parallel analysis of angiosperm *CCA1*/*LHY *genes, and their observations are consistent with those obtained in their analysis of the *PRR *genes; the common ancestor of monocots and eudicots had one *CCA1*/*LHY *gene and there has been independent duplication of the *LHY*/*CCA1 *genes in the monocots and eudicots. Within the eudicots, there has been independent duplication in poplar and *Arabidopsis*.

The key conclusion from these studies is that the common ancestor of the monocots and eudicots had the basic components necessary for the construction of a circadian clock with multiple interlocked feedback loops prior to the separation of these groups 200 million years ago [2]. This makes it very likely that the *Arabidopsis *clock will prove a useful model for most agricultural species. It will be interesting to determine whether the more basal angiosperms, such as the Magnoliales, also share this common clock architecture.

## Sub- and neo-functionalization among clock genes

**Figure 2:**
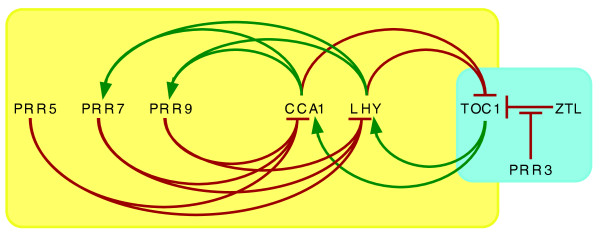
**Regulatory relationships among *TOC1*/*PRR*s and *CCA1*/*LHY***. CCA1, LHY, TOC1, PRR9, PRR7, and PRR5 all are recruited to promoters and regulate transcription (yellow shading). PRR9, PRR7, and PRR5 are negative regulators (indicated in red) of *CCA1 *and *LHY*, whereas CCA1 and LHY are both negative regulators (in red) of *TOC1 *and positive regulators (indicated in green) of *PRR7 *and *PRR9*. PRR3 is not known to regulate transcription. Instead (blue shading), PRR3 interacts with TOC1 protein to protect it from ZTL-mediated recruitment for proteasomal degradation. Modified from [10].

One consequence of gene duplication is that it allows the two copies to subdivide the functions of the ancestral copy (functional specialization or sub-functionalization), or for one copy to acquire a new function (neo-functionalization) while the other retains the original function, thus preserving fitness; but is there evidence for either functional specialization or acquisition of novel functions among *PRR *genes during evolution of the angiosperms? The strongest evidence comes from *Arabidopsis*, where clock function is best studied. *TOC1 *and four other *PRR *genes each show circadian oscillations in transcript abundance, with peak abundance occurring at intervals spanning the day starting at dawn with *PRR9*, followed by *PRR7*, *PRR5*, *PRR3*, and finally at dusk with *TOC1 *(*PRR1*) [4,5]. As shown in Figure 2, TOC1 is recruited to the *CCA1 *promoter and is a positive regulator of *CCA1 *expression, although the molecular details remain incompletely described [1]. PRR9, PRR7, and PRR5 are recruited to the promoters of *CCA1 *and *LHY *and negatively regulate their expression [10]. It is likely that the sequential expression of *PRR9*, *PRR7*, and *PRR5 *contributes to sustained repression of *CCA1 *and *LHY *expression throughout the day. This indicates that, while the function of these three genes is partially redundant, with normal expression of the three genes the temporal window of *CCA1*/*LHY *repression is extended. Thus *PRR9*, *PRR7*, and *PRR5 *offer an example of sub-functionalization in the temporal domain. Although the function of the rice (*Oryza sativa*) orthologs of *PRR9*, *PRR7*, and *PRR5 *has not been established, there is a similar sequential pattern of expression of *OsPRR73*/*OsPRR37 *and then *OsPRR95*/*OsPRR59*, followed by *OsTOC1 *(*OsPRR1*) [11].

In *Arabidopsis*, the *PRR3 *gene offers an example of acquisition of a novel function. *PRR9*, *PRR7*, and *PRR5 *all have a similar role in negatively regulating *CCA1 *and *LHY*, suggesting that this represents the ancestral function (Figure 2). *PRR3 *appears, instead, to have acquired a novel and specialized function in the vascular tissue, where PRR3 binds to TOC1 and, in doing so, blocks the interaction of TOC1 with ZTL, the F-box protein that targets TOC1 for proteasomal degradation [12]. Thus, *PRR3 *exhibits a restricted domain of expression and has acquired a novel function, the regulation of TOC1 stability through protein-protein interaction (Figure 2). In *Arabidopsis*, loss of *PRR3 *function confers only a very small shortening of circadian period [13], which is consistent with the apparent loss of *PRR3 *in poplar, without concomitant perturbation of clock function.

There are additional suggestions of evolving function in the *PRR7 *lineage. In *Arabidopsis*, *PRR7 *contributes to the determination of flowering time, although the effects are not large and *PRR7 *is not a major determinant of flowering time among natural populations [14]. In contrast, in the monocots barley and wheat, *PRR7 *(*Ppd-H1 *and *Ppd-D1*, respectively) is one of the major determinants of photoperiod sensitivity and flowering time [15,16]. Whether this represents a true acquisition of novel function in the monocots or a loss of function in the eudicots remains uncertain and will require more detailed dissection of the roles of *PRR7 *in the flowering pathways of monocots and eudicots.

## Future directions

There remains a great deal of work to achieve a mechanistic understanding of how the circadian clock keeps time. Four of the five PRR proteins are recruited to DNA yet they do not possess recognized DNA-binding domains and are not known to bind DNA directly. How are they recruited to the *CCA1 *and *LHY *promoters and what makes TOC1 a positive regulator while PRR5, PRR7, and PRR9 are repressors? Takata *et al. *[2,9] establish that the common ancestor of monocots and eudicots had *PRR *and *CCA1*/*LHY *genes and, therefore, the materials with which to construct a functional circadian clock. How has the differential amplification of these two gene families in the angiosperm lineages allowed modulation of circadian timekeeping? How well does the outline presented in Figures 1 and 2 apply across the angiosperms and to more primitive plants? Within species, has variation among clock genes contributed to fitness? There is no shortage of questions and the increasing availability of genome sequences and tools to probe gene function in many species make this a wonderful time to study the basis of circadian timing.
